# Robust orienting to protofacial stimuli in autism

**DOI:** 10.1016/j.cub.2013.10.034

**Published:** 2013-12-16

**Authors:** Punit Shah, Anne Gaule, Geoffrey Bird, Richard Cook

**Affiliations:** 1 Department of Psychology, City University, London EC1R OJD, UK; 2 Social, Genetic and Developmental Psychiatry Centre (MRC), Institute of Psychiatry, King’s College London, SE5 8AF, UK; 3 Division of Psychology and Language Sciences, University College London, WC1N 3AR, UK; 4 Institute of Cognitive Neuroscience, University College London, WC1N 3AR, UK

## Abstract

Newborn infants exhibit a remarkable tendency to orient to faces. This behavior is thought to be mediated by a subcortical mechanism tuned to the protoface stimulus: a face-like configuration comprising three dark areas on a lighter background. When this unique stimulus translates across their visual field, neurotypical infants will change their gaze or head direction to track the protoface [1–3]. Orienting to this low spatial frequency pattern is thought to encourage infants to attend to faces, despite their poor visual acuity [2,3]. By biasing the input into the newborn’s visual system, this primitive instinct may serve to ‘canalize’ the development of more sophisticated face representation. Leading accounts attribute deficits of face perception associated with Autism Spectrum Disorders (ASD) [4] to abnormalities within this orienting mechanism. If infants who are later diagnosed with ASD exhibit reduced protoface orienting, this may compromise the emergence of perceptual expertise for faces [5]. Here we report a novel effect that confirms that the protoface stimulus captures adults’ attention via an involuntary, exogenous process (Experiment 1). Contrary to leading developmental accounts of face perception deficits in ASD, we go on to show that this orienting response is intact in autistic individuals (Experiment 2).

## Main Text

Protoface orienting plays a critical role in the development of infants’ face perception and wider socio-cognitive abilities; however, the subcortical mechanism responsible is also thought to influence the behavior of adults [2]. Unlike most other visual stimuli, the protoface remains detectable by adults in continuous flash suppression paradigms, in which the input into one eye is typically rendered unperceivable by a stream of constantly changing input to the other eye [6]. This advantage disappears when the pattern is presented upside-down or in negative polarity. Similarly, when instructed to orient toward stimuli displayed peripherally, adults’ saccadic reaction times (RTs) to the protoface are faster than to orientation-inverted and polarity-reversed control patterns [7]. The difference between detecting, and orienting to, the protoface is not trivial; only orienting behaviors bias the input into the developing visual system and thereby canalize the emergence of sophisticated face representation. Nevertheless, the orientation and contrast specificity of the detection [6] and instructed orienting [7] effects seen in neurotypical adults resembles closely the exogenous orienting responses seen in neurotypical infants [1]. These effects may therefore depend on a common mechanism, mediated by subcortical structures (amygdala, superior colliculus, pulvinar), which is both present in neonates and persists into adulthood (see [2]).

**Figure 1:**
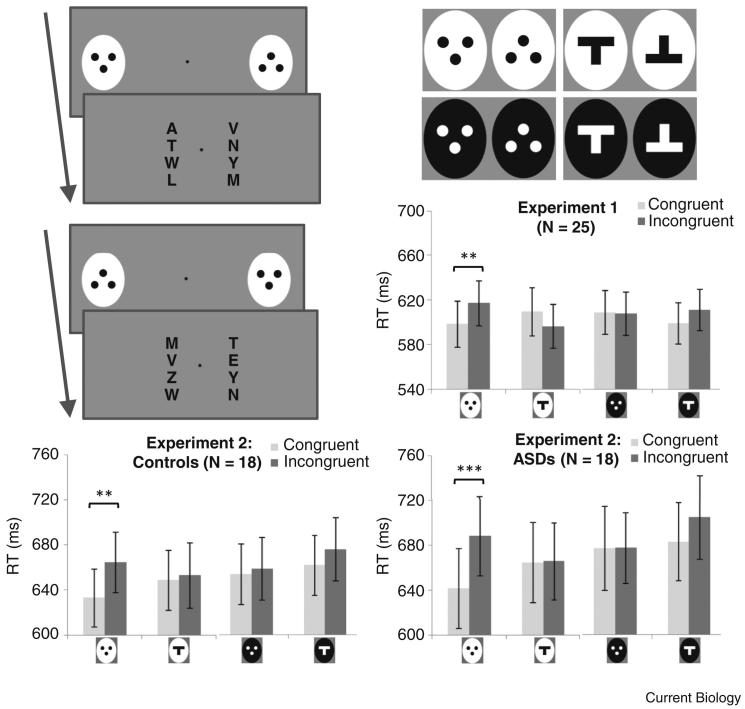
Experimental results with the attentional-cueing paradigm. Experimental trials required participants to indicate as quickly as possible which of two letter arrays, presented either side of fixation, contained a target letter (‘W’). Immediately before the onset of the arrays, the protoface and an inverted control pattern were presented at peripheral left and right locations, for 200 msec. Participants responded faster on congruent trials (left-top; protoface cued the correct location) than on incongruent trials (left-middle; protoface cued the incorrect location). Concurrent presentation of the inverted control pattern ensured that cueing effects were not due to low-level stimulus features (for example, luminance, contrast, edge). In close accordance with infant orienting responses [1], the cueing effect was selective for the protoface; other stimulus combinations (right-top) failed to yield significant congruency effects (right-middle). Contrary to leading accounts of face perception deficits in ASD [5], autistic individuals and matched neurotypical controls exhibited equivalent orienting responses (right-bottom and left-bottom, respectively). Significance at *p* < 0.010 is denoted by ^∗∗^; significance at *p* < 0.001 is denoted by ^∗∗∗^. (See also Table S1 in the Supplemental Information.)

In our first experiment, 25 neurotypical participants completed a novel attentional-cueing paradigm during which they were tasked with indicating, as quickly as possible, whether a target letter (‘W’) appeared in a left or right array (Figure 1, left-top, left-middle). Participants’ RTs (Figure 1; right-middle) were significantly faster (*t*(24) = 2.983, *p =* 0.006) when the correct side of the display was cued by presentation of the protoface (congruent trials) than when the protoface cued the incorrect side (incongruent trials). Contrary to the suggestion that orienting may be elicited by top-heavy patterns [8], no congruency effects (all *p* > 0.090) were observed for the protoface shown in negative polarity, or a T-pattern in either positive or negative polarity (Figure 1, right-top). This pattern accords with previous findings from eye-tracking procedures where participants were instructed to attend to protofacial stimuli [7]. Crucially, however, because the protoface stimulus is wholly task-irrelevant in the present paradigm, this result provides the first evidence that adults exhibit involuntary, exogenous orienting; the protoface captures attention despite being unrelated to the instructed task.

Our second experiment compared the performance of 18 adults with an ASD and 18 matched controls (see Supplemental Information) on this attentional cueing procedure. The control group demonstrated the same pattern of results seen in Experiment 1 (Figure 1, left-bottom), with significantly faster RTs (*t*(17) = 3.209, *p =* 0.005) on congruent trials than on incongruent trials. Critically, the ASD group showed the same pattern of results (Figure 1, right-bottom), demonstrating significantly faster RTs (*t*(17) = 4.851, *p <* 0.001) on congruent trials than on incongruent trials, indicative of robust orienting to the protoface. Neither group showed significant congruency effects for any of the control patterns (all *p* > 0.14). No group differences were seen in the orienting response to the protoface (*t*(34) = 1.121, *p* = 0.27) or to the control patterns (all *p* > 0.60). No association was observed between autism severity and protoface orienting (*r* = 0.044, *p* = 0.86). Furthermore, across all participants, no association was observed between autistic traits and orienting towards the protoface (r = –0.015, *p* = 0.93).

Leading accounts of the face processing deficits characteristic of ASD propose that faces are less able to capture the attention of autistic individuals because of abnormalities within a subcortical orienting mechanism. If, as a result, infants who later develop autism spend less time looking at faces, they may fail to develop equivalent perceptual expertise for faces, with distal consequences for related socio-cognitive functioning [5]. Contrary to these accounts, however, we find that individuals with ASD exhibit entirely typical orienting responses to the protoface; the stimulus thought most effective in recruiting the subcortical orienting mechanism [2]. The present results therefore speak against developmental accounts of ASD that invoke deficits in facial orienting [5]. Instead, this conclusion accords with recent evidence that children with ASD (5–12 years) display broadly age-appropriate orienting to photographs of adult faces [9].

Where observed, the failure of autistic individuals to develop typical perceptual expertise for faces may be better explained by a reduced propensity to maintain facial fixation as a result of diminished motivation [1,10]. The maintenance of facial fixation, following initial orienting, is controlled by a voluntary, endogenous process — we can choose to maintain attention or to look away. If individuals with ASD find social stimuli less rewarding, they may exhibit shorter fixations despite robust orienting responses. Shorter fixation durations — particularly if seen during critical periods of development — may reduce the fidelity with which faces are processed, thereby affecting the emergence of perceptual expertise with faces. Nevertheless, while differences in fixation maintenance remain a possibility, the present results indicate that the involuntary, exogenous orienting instinct thought crucial for perceptual and socio-cognitive development is intact in autism.

## Supplemental Information

Document S1. Experimental Procedures, Tables S1 and S2
