# Supplementary material for: Robust orienting to protofacial stimuli in autism
Source: Curr Biol. 2013 Dec 16;23(24):R1087–8. doi: 10.1016/j.cub.2013.10.034 (PMC3898081; doi:10.1016/j.cub.2013.10.034)
Supplement: Document S1. Experimental Procedures, Tables S1 and S2 [file mmc1.pdf]

**Supplemental Information: Robust orienting to protofacial stimuli in autism**

Punit Shah, Anne Gaule, Geoffrey Bird, & Richard Cook

## Experiment 1

### Supplemental Results

The RT data (Table S1) were analyzed using ANOVA with stimulus (protoface, T-pattern), polarity (positive, negative) and congruency (congruent, incongruent) as within-subjects factors. The analysis revealed a significant stimulus × polarity × congruency interaction [$F(1,24) = 8.392$, $p = .008$]. This interaction reflected the presence of a polarity × congruency interaction for the protoface [$F(1,24) = 11.275$, $p = .003$] but not the T-pattern [$F(1,24) = 2.109$, $p = .159$]. Crucially, RTs were significantly faster when the protoface cued the correct position when shown in positive polarity [$t(24) = 2.983$, $p = .006$], whilst no congruency effect was seen for the negative polarity protoface [$t(24) = 1.738$, $p = .095$].

### Supplemental Experimental Procedures

Twenty-five right-handed adults (8 male) participated in Experiment 1 ($M_{age}$ = 24.88 years, $SD_{age}$ = 5.67 years). All participants had normal or corrected-to-normal vision, gave informed consent, were paid for their participation, and were fully debriefed upon task completion. Ethical clearance was granted by the local ethics committee and the study was conducted in accordance with the ethical standards laid down in the 1964 Declaration of Helsinki.

The protoface stimulus and control patterns subtended 4° × 3° of visual angle when viewed at a distance of 60cm. Upright and inverted patterns were presented 12° apart. The aspect-ratio of the ‘T’ element was matched to the spatial extent of the protoface elements. Each letter array comprised 4 letters presented in white Arial font. The seven distractor letters were chosen from ‘A’ ‘E’ ‘F’ ‘H’ ‘K’ ‘L’ ‘M’ ‘N’ ‘V’ ‘X’ ‘T’ ‘Y’ ‘Z’ and presented at randomized locations. The target letter (‘W’) was equally likely to appear at each of the 8 locations in arrays. The arrays were presented 6° apart. The display background was grey (128 on a 0–255 scale); equidistant between black (0) white (255). Experimental programs were written in MATLAB using Psychtoolbox [S1, S2]. Stimuli were presented on a Dell LCD monitor at 60-Hz refresh rate.

Participants completed 6 practice trials before starting the experimental procedure, comprising 320 trials, grouped into 4 blocks of 80 trials. Overall the procedure lasted approximately 20 minutes. Trial type (positive polarity protoface; negative polarity protoface; positive polarity T-pattern; negative polarity T-pattern) was interleaved within each block. Participants were instructed to fixate on the central dot; to disregard all peripheral stimuli; and to respond as quickly as they could without sacrificing accuracy. Participants used the left and right arrow keys to record the array in which the target letter appeared. Reaction times (RTs) were taken to be the interval from the onset of the letter arrays, to the moment the participant responded. Mean RTs for each condition were calculated having excluded trials where participants made errors – where the location of the target letter was misidentified – or took longer than 1600 msecs to respond. In total, 3.46% of data points were lost; 3.07% and 0.39%, due to errors and slow responding, respectively. Analyses were conducted on the resulting RT distributions.

## Experiment 2

### Supplemental Results

The RT data (Table S1) were analyzed using ANOVA with stimulus (protoface, T-pattern), polarity (positive, negative) and congruency (congruent, incongruent) as within-subjects factors, and group (control, ASD) as a between-subjects factor. Crucially, this analysis revealed no main effect of ($p$ = .67), or interactions with (all $p$ > .15), group. Both the control and the participants with ASD showed significant stimulus × polarity × congruency interactions, [$F(1,17)$ = 8.017, $p$ = .012] and [$F(1,17)$ = 17.431, $p$ = .001], respectively. As in Experiment 1, these effects reflected the presence of polarity × congruency interactions for the protoface only, [$F(1,17)$ = 7.354, $p$ = .015] and [$F(1,17)$ = 18.641, $p$ < .001], respectively. The RTs of both the controls [$t(17)$ = 3.209, $p$ = .005] and the participants with ASD [$t(17)$ = 4.851, $p$ < .001] were significantly faster only when the correct location was cued by the protoface shown in positive polarity.

No association was observed between autism severity – as measured by the Autism Diagnostic Observation Schedule—Generic (ADOS-G; [S3]) – and orienting towards the protoface [$r$ = .044, $p$ = .86], the protoface in negative polarity [$r$ = -.084, $p$ = .74], the T-pattern in positive polarity [$r$ = -.26, $p$ = .30], or the T-pattern in negative polarity [$r$ = -.15, $p$ = .55]. Similarly, across all participants, no association was observed between autistic traits – as measured by the Autism-Spectrum Quotient (AQ; [S4]) – and orienting towards the protoface [$r$ = -.015, $p$ = .93], the protoface in negative polarity [$r$ = .079, $p$ = .65], the T-pattern in positive polarity [$r$ = -.067, $p$ = .70], or the T-pattern in negative polarity [$r$ = -.124, $p$ = .47].

Comparison of the data from Experiment 1 and Experiment 2 revealed significantly slower RTs across all conditions [$t(58.734) = 2.085$, $p = .041$] in Experiment 2 ($M = 665.52$ msec, $SD = 129.30$ msec) than in Experiment 1 ($M = 605.52$ msec, $SD = 95.48$ msec). This almost certainly reflects the fact the participants in Experiment 2 were older ($M_{age} = 41.0$ years) than in Experiment 1 ($M_{age} = 24.9$ years). Consistent with the widely accepted view that slower RTs are seen in older populations [e.g., S5], a significant correlation was observed between age and average RT ($r = .523$, $p < .001$). Nevertheless, no association was seen between age and the degree of the protoface orienting i) across all 61 participants [$r = -.081$, $p = .54$], ii) in the 43 typical participants' [$r = -.169$, $p = .28$] or iii) in the participants with ASD [$r = -.211$, $p = .40$]. Similarly, no association was seen between mean RT across trials and the degree of the protoface orienting i) across all 61 participants [$r = .087$, $p = .505$], ii) in the 43 typical participants' [$r = .073$, $p = .641$] or iii) in the participants with ASD [$r = -.012$, $p = .96$].

**Supplemental Experimental Procedures**

Thirty-six right-handed adults with ($n = 18$) and without autism ($n = 18$) participated in Experiment 2. Participants with autism spectrum disorders (ASD) were recruited from a database held at the Institute of Cognitive Neuroscience at University College London. All had received independent clinical diagnosis (according to the DSM-IV; [S6]) of an ASD from a clinical practitioner. All participants also met the criteria for autism or autism spectrum disorder on the ADOS-G [S3]. All participants completed the AQ [S4] to measure autistic traits, for which the ASD group scored significantly higher than the control group (see Table S2). Finally, all 36 participants reported normal or corrected-to-normal vision.

The stimuli and procedure used in Experiment 2 were identical to those of Experiment 1. As in the first experiment, mean RTs were calculated having first excluded errors and responses exceeding 1600 msec. Overall 3.03% of data points were lost from the control group (2.22% and 0.81% due to errors and slow responding) and 2.22% were lost from the ASD group (1.38% and 0.84% due to errors and slow responding). There were no group differences in the number of trials omitted from analysis, due to either responding too slowly ($p$ = .93) or incorrectly ($p$ = .21).

**Table S1:** Mean RTs (msecs) observed in Experiment 1 & 2. Standard deviations are shown in italics inside parentheses.

| | | | Protoface | | T-Pattern | |
|---|---|---|---|---|---|---|
| | | **Polarity** | Positive | Negative | Positive | Negative |
| **Experiment 1** | | **Congruent** | 598 (*103*) | 609 (*100*) | 608 (*98*) | 599 (*97*) |
| | | **Incongruent** | 617 (*108*) | 596 (*98*) | 607 (*92*) | 610 (*91*) |
| **Experiment 2** | **Control group** | **Congruent** | 633 (*108*) | 649 (*113*) | 654 (*114*) | 661 (*117*) |
| | | **Incongruent** | 664 (*103*) | 653 (*122*) | 658 (*112*) | 676 (*119*) |
| | **ASD group** | **Congruent** | 641 (*150*) | 665 (*150*) | 677 (*158*) | 683 (*134*) |
| | | **Incongruent** | 688 (*152*) | 666 (*146*) | 677 (*148*) | 704 (*158*) |

**Table S2**: Mean Age, Gender, Autism-Spectrum Quotient (AQ) and IQ scores [S7] for the autism spectrum disorders (ASD) group and the matched neurotypical controls. Autism Diagnostic Observational Schedule (ADOS) score and classification details for the ASD group. Standard deviations are shown in italics inside parentheses.

| | **ASD** | **Controls** | **Comparison** |
|---|---|---|---|
| *N* | 18 | 18 | - |
| Gender | 16 Male, 2 Female | 16 Male, 2 Female | - |
| Mean Age (Years) | 40.72 (*11.90*) | 41.33 (*13.45*) | *p* = .886 |
| Mean Full-scale IQ | 115.39 (*10.00*) | 112.11 (*13.59*) | *p* = .416 |
| Mean AQ | 34.50 (*8.84*) | 15.06 (*6.03*) | *p* < .001 |
| ADOS Classification | 11 Autism, 7 Autism Spectrum | - | - |
| Mean ADOS-G Score | 10.22 (*2.69*) | - | - |

*Note*. ADOS-G score is derived from a diagnostic algorithm [S1] with a higher score representing a higher degree of autism.

## Supplemental References


S1. Brainard, D.H. (1997). The psychophysics toolbox. Spat. Vis. *10*, 433-436.

S2. Pelli, D.G. (1997). The VideoToolbox software for visual psychophysics: transforming numbers into movies. Spat. Vis. *10*, 437-442.

S3. Lord, C., Risi, S., Lambrecht, L., Cook, E.H., Jr., Leventhal, B.L., DiLavore, P.C., Pickles, A., and Rutter, M. (2000). The autism diagnostic observation schedule-generic: a standard measure of social and communication deficits associated with the spectrum of autism. J. Autism Dev. Disord. *30*, 205-223.

S4. Baron-Cohen, S., Wheelwright, S., Skinner, R., Martin, J., and Clubley, E. (2001). The autism-spectrum quotient (AQ): evidence from Asperger syndrome/high-functioning autism, males and females, scientists and mathematicians. J. Autism Dev. Disord. *31*, 5-17.

S5. Ratcliff, R., Thapar, A., and McKoon, G. (2001). The effects of aging on reaction time in a signal detection task. Psychol. Aging *16*, 323-341.

S6. American Psychiatric Association. (1994). Diagnostic and Statisitcal Manual of Mental Disorders (DSM-IV), (Washington, DC: Author).

S7. Wechsler, D. (1997). Wechsler Adult Intelligence Scale (3rd Edition), (San Antonio, TX: Psychological Corporation).
